# G-rich motifs within phosphorothioate-based antisense oligonucleotides (ASOs) drive activation of *FXN* expression through indirect effects

**DOI:** 10.1093/nar/gkac1108

**Published:** 2022-12-13

**Authors:** Feng Wang, Ezequiel Calvo-Roitberg, Julia M Rembetsy-Brown, Minggang Fang, Jacquelyn Sousa, Zachary J Kartje, Pranathi Meda Krishnamurthy, Jonathan Lee, Michael R Green, Athma A Pai, Jonathan K Watts

**Affiliations:** RNA Therapeutics Institute, University of Massachusetts Chan Medical School, Worcester, MA, 01605, USA; RNA Therapeutics Institute, University of Massachusetts Chan Medical School, Worcester, MA, 01605, USA; RNA Therapeutics Institute, University of Massachusetts Chan Medical School, Worcester, MA, 01605, USA; Department of Molecular, Cell and Cancer Biology, University of Massachusetts Chan Medical School, Worcester, MA, 01605, USA; RNA Therapeutics Institute, University of Massachusetts Chan Medical School, Worcester, MA, 01605, USA; RNA Therapeutics Institute, University of Massachusetts Chan Medical School, Worcester, MA, 01605, USA; RNA Therapeutics Institute, University of Massachusetts Chan Medical School, Worcester, MA, 01605, USA; RNA Therapeutics Institute, University of Massachusetts Chan Medical School, Worcester, MA, 01605, USA; Department of Molecular, Cell and Cancer Biology, University of Massachusetts Chan Medical School, Worcester, MA, 01605, USA; RNA Therapeutics Institute, University of Massachusetts Chan Medical School, Worcester, MA, 01605, USA; RNA Therapeutics Institute, University of Massachusetts Chan Medical School, Worcester, MA, 01605, USA; Department of Biochemistry and Molecular Biotechnology, University of Massachusetts Chan Medical School, Worcester, MA, 01605, USA

## Abstract

Friedreich’s ataxia is an incurable disease caused by frataxin (FXN) protein deficiency, which is mostly induced by GAA repeat expansion in intron 1 of the *FXN* gene. Here, we identified antisense oligonucleotides (ASOs), complementary to two regions within the first intron of *FXN* pre-mRNA, which could increase *FXN* mRNA by ∼2-fold in patient fibroblasts. The increase in *FXN* mRNA was confirmed by the identification of multiple overlapping *FXN*-activating ASOs at each region, two independent RNA quantification assays, and normalization by multiple housekeeping genes. Experiments on cells with the ASO-binding sites deleted indicate that the ASO-induced *FXN* activation was driven by indirect effects. RNA sequencing analyses showed that the two ASOs induced similar transcriptome-wide changes, which did not resemble the transcriptome of wild-type cells. This RNA-seq analysis did not identify directly base-paired off-target genes shared across ASOs. Mismatch studies identified two guanosine-rich motifs (CCGG and G_4_) within the ASOs that were required for *FXN* activation. The phosphorodiamidate morpholino oligomer analogs of our ASOs did not activate *FXN*, pointing to a PS-backbone-mediated effect. Our study demonstrates the importance of multiple, detailed control experiments and target validation in oligonucleotide studies employing novel mechanisms such as gene activation.

## INTRODUCTION

Friedreich’s ataxia (FA) is caused by a deficiency of the mitochondrial protein frataxin, expressed from the *FXN* gene. There are approximately 15 000 FA patients worldwide, and the lack of any disease-modifying therapeutic options results in poor quality of life and shortened life span (average 40–50 years) (1). Approximately 96% of FA cases are caused by a GAA trinucleotide repeat expansion in the intron 1 of both alleles of *FXN* (2). The current FA treatment pipeline covers a wide range of strategies, including improving mitochondrial function, reducing oxidative stress, modulating *FXN*-controlled pathways and increasing *FXN* expression by various modalities (3).

Oligonucleotides are emerging as a promising modality for treating neurological disorders (4). Most of the compounds in clinical development are based on gene silencing or splice switching. Multiple oligonucleotide-based activation mechanisms for gene activation have also been proposed, including promoter-targeted duplex RNAs (5–8), inhibition of repressive antisense transcripts (9), reducing noisy splicing (10,11), stabilizing mRNAs (12), enhancement of translation (13–16) and reducing nonproductive translation initiation (17). But to date, gene activation by oligonucleotides has proven to be significantly more challenging than gene silencing. As a result, most gene-activating oligonucleotides are at the preclinical stage (4).

Studies have shown that antisense oligonucleotides (ASOs) targeting the *FXN* transcript within the expanded GAA repeat can restore *FXN* expression in cells (18–21). However, these compounds have not shown activity in mouse models of FA (22), and there are at least theoretical risks of off-target events inherent to the GAA-repeat-targeting strategy.

Here, we set out to develop non-repetitive ASOs that activate *FXN* expression by targeting within the first intron of the *FXN* gene. We identified two hotspots (S10 and S30) for activation, and in each case identified multiple overlapping ASOs able to increase *FXN* mRNA expression by ∼2-fold in patient-derived fibroblasts GM03816 (330, 380 GAA). Sequence and length optimization of the ASOs yielded additional hits that consistently activated *FXN* expression. We verified by multiple normalization controls and two independent RNA quantification assays (RT-qPCR and QuantiGene assay) that the activation was not a normalization artifact. The *FXN* activation by S10 and S30 was consistent in multiple cell models including patient-derived fibroblasts GM04078 (420, 541 GAA) as well as wild-type (WT) fibroblasts and other WT cells including HEK 293T, JHH2 and U87 cells. Thus, the activation was independent of GAA-repeat length. ASOs S10 and S30 were unable to activate *FXN* expression in FA mouse models.

RNA sequencing (RNA-seq) revealed that S10- and S30-derived ASOs could drive a unique transcriptomic profile in GM38016 cells, which did not resemble that of WT fibroblasts. Subsequent bioinformatic analyses did not identify base-paired off-target genes shared across the ASOs. However, deletion of the ASO-binding sites from the genome demonstrated that the *FXN* activation by S10- and S30-derived ASOs was mediated by an indirect effect. We then identified two guanosine-rich motifs (CCGG and G_4_) within the ASOs that were required for *FXN* activation in a mismatch study. The phosphorodiamidate morpholino oligomer (PMO) analogs of S10 and S30 ASOs did not activate *FXN*, indicating a PS-backbone-mediated effect. Taken together, these evidences point to the hypothesis that the activation, we observed, may be mediated by protein binding to guanosine-rich motifs within PS-modified ASOs rather than base pairing to *FXN* mRNA or to other transcripts.

Our study demonstrates the importance of multiple, detailed control experiments and target validation, which can minimize the risk of advancing false positives into further development, thus improving the ultimate success rate in developing oligonucleotide drugs, particularly those utilizing novel mechanisms such as activating gene expression.

## MATERIALS AND METHODS

### Oligonucleotide synthesis

Oligonucleotides were synthesized in house at a 1 μmol scale on a Biolytic Dr Oligo 48 synthesizer. Standard phosphoramidites were purchased from ChemGenes. Oxidation to phosphodiester linkages was accomplished with 0.05 M Iodine in 90% pyridine/10% water (ChemGenes, RN-2238). Sulfurization to phosphorothioate (PS) linkages was accomplished with 3-((dimethylamino-methylidene)amino)-3H-1,2,4-dithiazole-3-thione (DDTT), 0.1 M solution (ChemGenes, RN-1689). Oligonucleotides were deprotected in 30% NH_3_ in water (16 h at 55°C), and then the ammonia was removed under vacuum. The oligonucleotides were then desalted (3× RNase-free water wash, 15 min, 14 K rpm) using Amicon Ultra 0.5 ml 3 K filters (Millipore, UFC5003) and resuspended in 400 μl of RNase-free water.

Oligonucleotides were analyzed on an Agilent 6530 Q-TOF LC/MS system with electrospray ionization and time-of-flight ion separation in negative ionization mode. Data were analyzed using Agilent MassHunter software. Liquid chromatography was performed using a 2.1 × 50 mm AdvanceBio oligonucleotide column (Agilent Technologies, 659750–702). Buffer A: 100 mM hexafluoroisopropanol with 9 mM triethylamine in water. Buffer B: 100 mM hexafluoroisopropanol with 9 mM triethylamine in methanol. Samples were resolved over an elution gradient of 0–100% Buffer B over 5.5 min.

### Cell culture and *in vitro* screening

Fibroblast cells (Coriell Institute, GM03816 [330, 380 GAA] and GM04078 [420, 541 GAA]) and WT Primary Dermal Fibroblast (ATCC, PCS-201–012) were cultured in Dulbecco’s Modified Eagle’s Medium-high glucose (DMEM, D6429, MilliporeSigma) with 10% fetal bovine serum (FBS) and 1% nonessential amino acid. U87 and 293T were cultured in 90% DMEM and 10% FBS.

A total of 3000 fibroblast cells were seeded in each well of the 96-well plate and cultured at 37°C with 5% CO_2_. After 6–9 h, lipofectamine RNAiMAX (Invitrogen, 13778150) was used to transfect dsRNAs (25 nM) or ASOs (12.5 nM) with a 4-fold lower ratio of lipofectamine to oligonucleotide relative to the manufacturer’s recommended protocol. The cells were incubated with full media and transfection reagents for 72 h before RNA quantification.

### mRNA quantification by real-time quantitative PCR

RNA from cells and mouse tissues was extracted by RNeasy Plus Mini Kit (Qiagen, 74136), according to the manufacturer’s recommended protocol and measured by Nanodrop. Identical amount of RNA (1 μg) was used to generate cDNA by High-Capacity cDNA Reverse Transcription Kit (ThermoFisher, 4368814). qPCR was conducted using iTaq Universal SYBR Green Supermix (Bio-Rad, 1725121) with 25–50 ng of cDNA as input. qPCR cycling conditions and primers can be found in Supplementary Table S3.

### mRNA quantification by branched DNA (bDNA) assay

Branched DNA (bDNA) assay was performed using QuantiGene SinglePlex assay kit (ThermoFisher, QS0011) as previously described (23). In brief, cells were lysed in diluted lysis mixture containing 1 volume of lysis mixture (Invitrogen, 13228), 2 volumes of water and proteinase K (Invitrogen, 25530–049). Mouse brains were harvested and immediately sliced into 300 μm sections on a vibratome. Approximately 2 mm punches were taken from the coronal section of mid brain and put into RNAlater (Sigma, R0901). Tissues were lysed in homogenizing buffer (Invitrogen, QG0517) with proteinase K. bDNA probe sets can be found in Supplementary Table S3.

### Establishment of single clones with ASO-binding site deletion

293T cells were electroporated with Cas9 ribonucleoprotein (RNP) using Neon Transfection. Cas9 protein (IDT, 1081058) and sgRNA (IDT, Alt-R CRISPR-Cas9 sgRNA) were incubated at room temperature for 30 min, according to IDT’s recommendation. A total of 10^5^ 293T cells were electroporated Cas9 RNP complex (1700 V, 20 ms, 1 pulse). Single clones were seeded and genotyped as previously described (24). sgRNAs and genotyping primers can be found in Supplementary Table S3.

### Primary neuron isolation and culture

Mouse primary neurons were isolated and cultured as previously described (25). In brief, primary cortical neurons were isolated from E15.5 mouse embryos of pregnant mice and seeded in plating media on cell-culture plates coated with 0.01% Poly-L-Lysine. Plating media were replaced by feeding media 12 h after seeding the cells. Plating media: 500 ml of Neurobasal Plus (Gibco, A3653401) and 10 ml of B-27 Plus Supplement (Gibco, A3582801) with 2.5% FBS. Feeding media: plating media (without FBS), 4.8 μg/ml 5′UTP (Sigma, U6625) and 2.4 μg/ml 5′FdU (Sigma, F3503). Primary neurons were treated with ASOs for 7 days before harvest for RNA quantification.

### Intracerebroventricular (ICV) injection

Unilateral intracerebroventricular (ICV) injections were carried out under UMass Chan Medical School IACUC protocol A-2551 as previously described (26). In brief, YG8R mice at ∼12 weeks old were anesthetized by intraperitoneal injection of a sterile saline solution containing fentanyl/midazolam/dexmedetomidine (0.1, 5 and 0.25 mg/kg, respectively). ICV injection was performed at the following coordinates from bregma: 0.3 mm posterior, 1.0 mm dextrolateral and 3.0 mm ventral. Approximately 50 and 40 nmol ASO in 10 μl was injected in male and female YG8R mice, respectively. Brain tissues were harvested for analysis 8 days after ICV injection. We confirmed before carrying out the transgenic mouse studies that the primers/probes used were specific to human *FXN* (Supplementary Figure S2).

### RNA sequencing and analysis

Total RNA from three independent replicates following the indicated treatments 72 h post-transfection was extracted using TriZol reagent, according to the manufacturer’s protocol. mRNA was enriched using the Poly(A) mRNA Magnetic Isolation Module (New England Biolabs, 7490S). Sequencing libraries were prepared using the TruSeq Stranded Illumina Total RNA Preparation Kit (Illumina, 20020599) and sequenced in-house on an Illumina NextSeq550 machine with single-end 75 nt reads for approximately 25 M reads per sample. Data are available on the Gene Expression Omnibus under accession ID GSE205526.

Gene expression levels were estimated with kallisto (v 0.4.0) (27) using the hg38 reference genome (28) and ENSEMBL hg38.95 annotations to obtain transcripts per million (TPM). Reads were mapped using STAR (v2.7.0e) (29) and raw read counts were obtained using ht-seq (v0.10.0) (30). Differential gene expression analyses were performed with DESeq2 (v1.28.1) (31) using ht-seq output on genes with at least 10 reads in any sample. All differential expression analyses used ASO-NTC samples as controls.

To find potential off-target sites, we used a custom python script available at https://doi.org/10.5281/zenodo.7262358. Briefly, it scans input fasta files for sequences that are partially or fully complementary to the ASO sequence of interest. A pairing score was calculated using pairing values from a custom score matrix for each ASO. Details of the scoring matrices for each ASO can be found in Supplementary Table S5.

### Statistical analysis

The statistical analysis was performed in Prism software using one-way ANOVA with Dunnett correction for multiple comparisons relative to ASO-NTC or FA-UTC. Each data point in the figures represents the data from one independent biological replicate (one independent well of cells or one independent mouse). Error bars represent standard deviation.

## RESULTS

### ASOs targeting *FXN* intron 1 activate *FXN* expression in patient-derived fibroblasts

**Figure 1.**
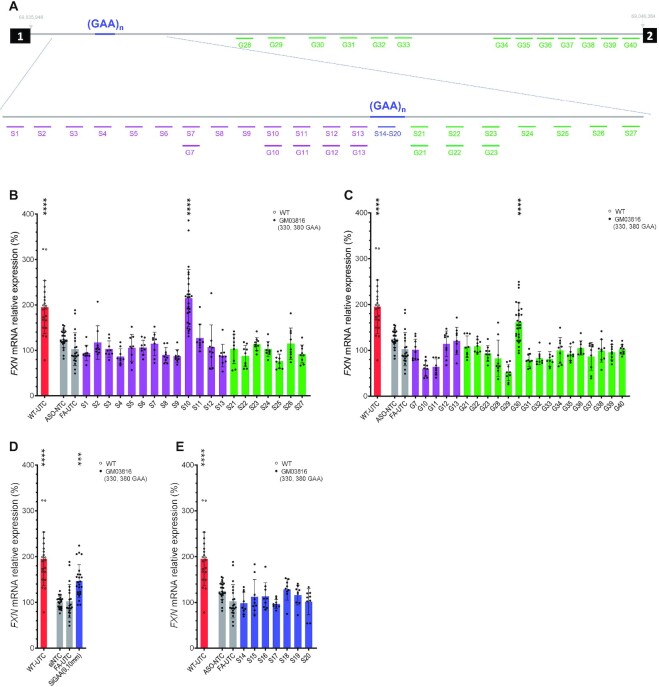
Design and screen of ASOs targeting intron 1 of *FXN* pre-mRNA. (**A**) Steric blocker and gapmer ASO design map. Numbered black boxes represent exons and the gray line represents intron 1. S1-S27 are steric blocker ASOs and G7-G40 are gapmer ASOs. (**B–E**) Oligonucleotide screen in GM03816 cells. *FXN* mRNA was quantified after 72 h treatment of steric blockers (B), gapmers (C), and repeat-targeted double-stranded RNA (dsRNA) and ASOs (D and E). All ASOs were transfected at 12.5 nM (B, C and E), and dsRNAs were transfected at 25 nM (D); WT-UTC, untreated WT fibroblasts (shown as a red bar with hollow symbols). All other bars represent experiments in GM03816 patient-derived fibroblasts (330, 380 GAA repeats). Gray bars represent negative controls (ASO-NTC, nontargeting control ASO; UTC, untreated cells [media only]). Magenta bars represent ASOs targeting upstream of the repeat, green bars downstream of the repeat, and blue bars within the repeat. *P* < 0.001 (***) and *P* < 0.0001 (****) were calculated relative to FA-UTC by one-way ANOVA.

FA is caused by an expanded GAA repeat within intron 1 of *FXN*. Previous work has shown that both steric blocker and gapmer ASOs designed to target the expanded GAA repeat can lead to activation of *FXN* expression (18–21). However, an NCBI BLAST search indicated 29 genes carrying at least 6 GAA repeats, suggesting that there are off-target risks of repeat-targeting ASOs (Supplementary Table S1). There is evidence that the expanded repeat may increase the R-loop formation into regions of the gene beyond the expanded repeat region itself (32). Therefore, we set out to design a series of ASOs binding proximal to the expanded repeat but in nonrepetitive regions of intron 1 (Figure 1A). We synthesized these compounds both as steric blockers (fully modified with 2′-*O*-methoxyethyl RNA [MOE] and with PS linkages) and MOE gapmers.

We treated patient-derived fibroblasts (GM03816 cells: 330, 380 GAA repeats) with ASOs at 12.5 nM, harvested RNA after 72 h and quantitated the *FXN* mRNA level using the QuantiGene bDNA assay. *FXN* expression is inherently variable (Supplementary Figure S1), requiring us to carry out these experiments at relatively large replicate numbers (16,18–21,33,34). Nevertheless, we were able to identify two clear hits in different regions of the intron. Steric blocker S10, targeting ∼240 bp upstream of GAA repeat and within the predicted R-loop region (32), showed a significant 2-fold increase in *FXN* mRNA (Figure 1B). Similarly, gapmer G30 targeting ∼4 kb downstream of GAA repeat also induced significant *FXN* activation (Figure 1C). For comparison, we also included WT fibroblasts cultured under identical conditions in the same batches of experiments and observed that both S10 and G30 were able to restore *FXN* expression similar to that in these WT cells (WT-UTC, Figure 1B and C).

We compared the activity of these hits to the previously identified repeat-targeted ASOs and double-stranded RNA (18,20). Our hits S10 and G30 showed more robust activation than the repeat-targeted compounds (Figure 1D–E).

### Sequence optimization of *FXN*-activating ASOs

**Figure 2.**
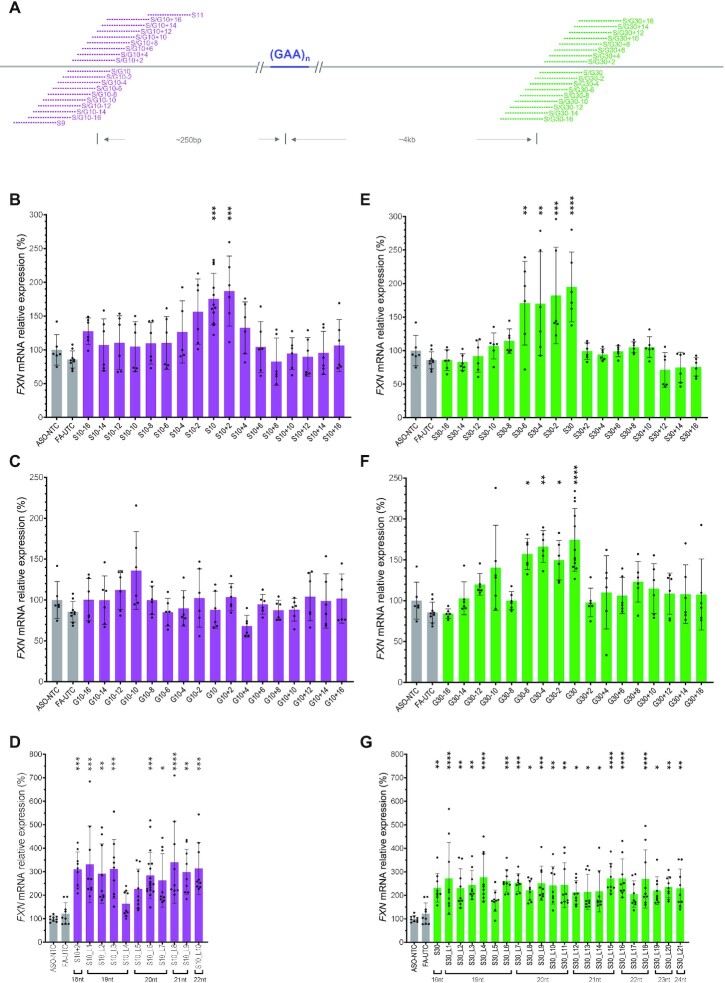
Micro-walk screen of S10 and G30 ASOs in GM03816 cells. (**A**) ASO micro-walk design map. (**B** and **C**) Micro-walk screen of S10 (steric blocker, B) and G10 (gapmer, C). (**D**) Screen of S10-based ASOs ranging from 18 to 22 nt. (**E** and **F**) Micro-walk screen of S30 (steric blocker, E) and G30 (gapmer F). (**G**) Screen of S30-based ASOs ranging from 18 to 24 nt. Gray bars represent negative controls (ASO-NTC: nontargeting control ASO. FA-UTC: untreated cells [media only]). Magenta represent ASOs targeting upstream of the repeat and green bars downstream of the repeat. *P* < 0.05 (*), *P* < 0.01 (**), *P* < 0.001 (***) and *P* < 0.0001 (****) were calculated relative to ASO-NTC by one-way ANOVA.

To learn whether additional ASOs overlapping the S10 and G30 sites could also activate *FXN* expression, we carried out a 2 nt micro-walk up to 16 bases in the 5′ and 3′ directions from both sites (Figure 2A). For each sequence in this study, we synthesized both steric blocker and gapmer ASOs. We observed that both S10 and S10+2 could achieve robust *FXN* activation (Figure 2B). The gapmer analogs G10 and neighboring sequences did not activate *FXN* expression (Figure 2C).

In the micro-walk from our second hit, ASOs G30-6, G30-4, G30-2 and G30 all could significantly increase *FXN* mRNA (Figure 2E). Interestingly, at this second site, we observed that the steric blocker ASOs S30-6, S30-4, S30-2 and S30 induced a similar level of *FXN* activation to their gapmer ASOs, demonstrating that RNA cleavage was not required for *FXN* activation (Figure 2F). Our previous work showed that gapmer ASOs might trigger more severe neurotoxicity in mouse brain than steric blocker ASOs targeting the same sequence (26). Thus, in moving forward, we focused our work on the steric blocker S30 at this region.

We further explored the targeting sequences by synthesizing ASOs of various lengths targeting S10 and S30 hotspots. ASOs of 18–22 nt targeting the S10 hotspot and ASOs of 18–24 nt targeting the S30 hotspot activated *FXN* expression to a similar extent (Figure 2D and G).

Overall, the fact that we found multiple active sequences of various lengths across two nonoverlapping hotspots suggested to us that *FXN* activation by these ASOs was likely a sequence specific on-target event.

### *FXN* activation at both hotspots is dose-responsive and not caused by normalization artifacts

**Figure 3.**
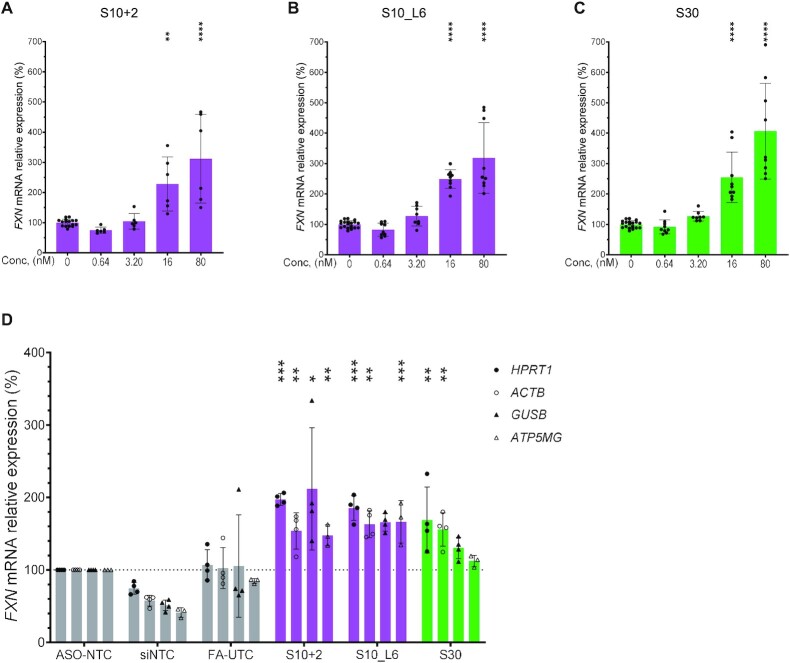
*FXN* activation by S10 and S30 is dose-responsive and consistent under normalization by multiple housekeeping genes. (**A–C**) Dose-responsive activation of *FXN* by S10+2 (A), S10_L6 (B), and S30 (C). (**D**) qPCR confirmation of *FXN* activation by S10 and S30 normalized to different housekeeping genes (*HPRT1*, *ACTB*, *GUSB* and *ATP5MG*). Gray bars represent negative controls (ASO-NTC, nontargeting control ASO; FA-UTC, untreated cells [media only]; siNTC, nontargeting siRNA). Magenta bars represent ASOs targeting upstream of the repeat and green bars downstream of the repeat. *P* < 0.05 (*), *P* < 0.01 (**), *P* < 0.001 (***) and *P* < 0.0001 (****) were calculated relative to 0 nM (in A–C) or to ASO-NTC (in D) by one-way ANOVA.

We further tested the *FXN*-activating ASOs at different concentrations, which showed dose-responsive activation (Figure 3A–C). Together with the data in previous screening (Figure 2B, D and E), we observed that all of the ASOs showed significant activation at 12.5 nM and above, but did not activate *FXN* expression at 3.2 nM or a lower concentration.

If compounds caused silencing of a normalization gene, this could be confused for activation leading to false positive results. To check whether the apparent increase of *FXN* mRNA by S10- and S30-derived ASOs could be a false positive due to effects on housekeeping gene expression, we quantified *FXN* mRNA level normalized by four different housekeeping genes using RT-qPCR. Even when normalized to most other housekeeping genes, the *FXN* activation by S10+2, S10_L6 and S30 was maintained, suggesting a real increase in *FXN* mRNA in cells treated by these ASOs instead of a normalization-driven false positive result (Figure 3D).

### *FXN* activation at both hotspots is independent of GAA repeat length

**Figure 4.**
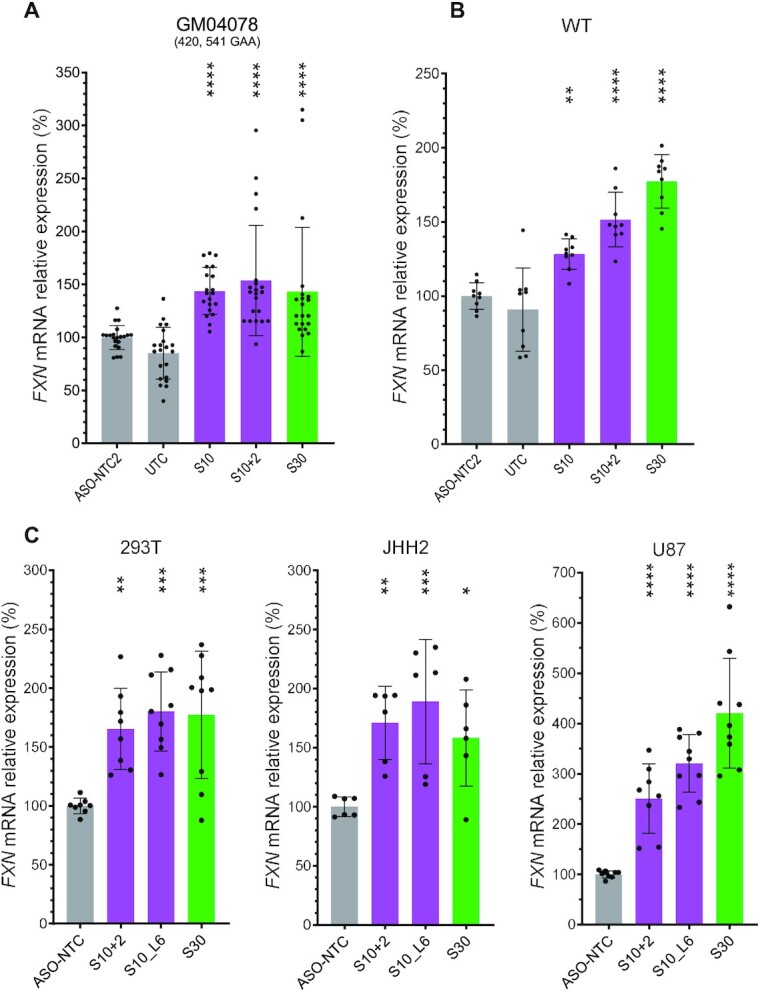
*FXN* activation by S10 and S30 is independent of GAA-repeat length. (**A–C**) Consistent *FXN* activation by S10 and S30 in GM04078 patient-derived fibroblasts with longer repeat length (A), WT fibroblasts (B) and nonfibroblast cells (293T, JHH2 and U87) (C). Gray bars represent negative controls (ASO-NTC and ASO-NTC2, nontargeting control ASOs; UTC, untreated cells [media only]). Magenta bars represent ASOs targeting upstream of the repeat and green bars downstream of the repeat. *P* < 0.05 (*), *P* < 0.01 (**), *P* < 0.001 (***) and *P* < 0.0001 (****) were calculated relative to the ASO-NTCs by one-way ANOVA.

To understand whether S10- and S30-derived ASOs can activate *FXN* expression in patient-derived cells with longer GAA repeats, we tested them in another patient-derived fibroblast, GM04078, which carries over 400 GAA repeats in each allele. Encouragingly, the ASOs achieved significant *FXN* activation in GM04078 cells, bringing the *FXN* mRNA level similar to that of untreated WT cells (WT-UTC control, Figure 4A).

To our surprise, the S10- and S30-derived ASOs could also increase *FXN* mRNA level in WT fibroblasts, indicating this ASO-induced *FXN* activation was independent of GAA repeat length (Figure 4B). We then tested these *FXN*-activating ASOs in more cell lines from various tissue origins including HEK-293T (kidney), JHH2 (hepatocellular carcinoma) and U87 (glioma) and observed significant *FXN* mRNA increase by S10+2, S10_L6 and S30 (Figure 4C). Thus, S10- and S30-derived ASOs can increase *FXN* mRNA expression in various cell types with distinct transcriptomic backgrounds.

### S10 and S30 cannot activate *FXN* expression in FA mouse models

**Figure 5.**
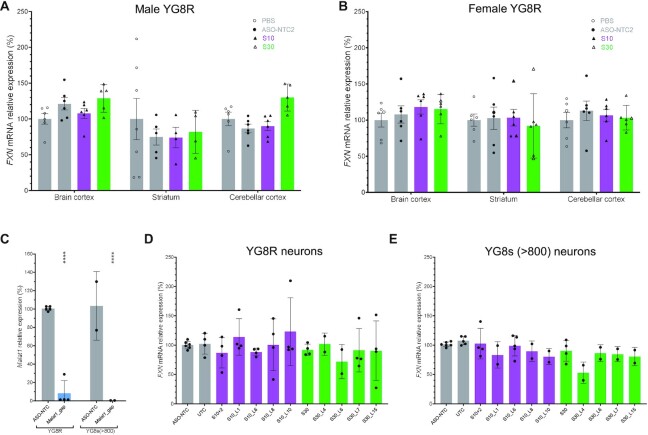
S10 and S30 cannot activate *FXN* expression in FA mouse model or primary mouse neurons. (**A** and **B**) *FXN* expression in indicated mouse brain regions of male (A) and female (B) YG8R mice after ICV injection. (**C**) Robust knockdown of *Malat1* RNA by *Malat1*_gap (1.5 μM) in primary neurons isolated from YG8R and Fxn^null^::YG8s(GAA)_>800_ fetuses [abbreviated as YG8s (>800)]. (**D** and **E**) *FXN* expression in primary neurons established from YG8R (D) and YG8s(>800) (E) after 1.5 μM ASO treatment. The genotypes of primary neurons were *Fxn*^−/−^, Tg^+/+^ in (C–E); Tg, transgene.

To test the *FXN*-activating ASOs *in vivo*, we used the YG8R mouse model, which had both mouse *Fxn* alleles knocked out and carried two tandem copies of the human *FXN* gene with ∼82 and ∼190 GAA repeats (35). Eight days after ICV injection of ASOs, the *FXN* mRNA quantification showed no difference between the control group and the ASO group in various brain regions (Figure 5A and B).

We wanted to explore whether this negative result reflected insufficient pharmacokinetics of the ASOs, different response to ASOs in the various cell types in the brain or a fundamental inability to activate in neurons from this mouse model. Therefore, we tested the *FXN*-activating ASOs in primary embryonic neurons from the breeding of YG8R (Figure 5D) and *Fxn*^null^::YG8s(GAA)_>800_ (Figure 5E) (36). A gapmer targeting mouse *Malat1* showed >90% silencing of *Malat1* RNA, indicating successful delivery in the *in vitro* primary neuron system (Figure 5C). However, neither S10- or S30-derived ASOs showed significant *FXN* activation in these primary neurons (Figure 5D–E). We considered that might be due to species differences in the essential factors involved in *FXN* activation observed in human cell models, and it highlighted to us the need to understand the mechanism of *FXN* activation that we observed in human cell models.

### *FXN* activation by S10- and S30-derived ASOs is independent of RNase H1 function

**Figure 6.**
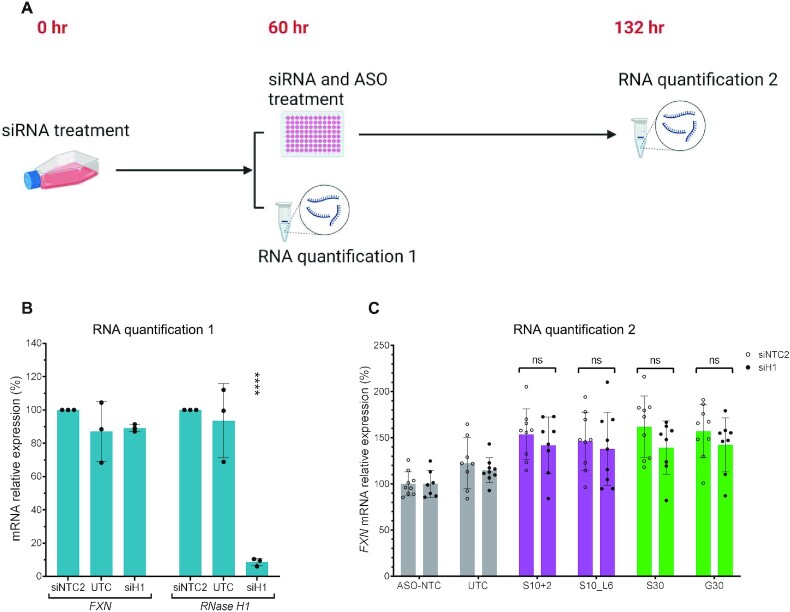
*FXN* activation by S10 and S30 is independent of RNase H1 function. (**A**) Experimental design investigating the dependence on RNase H1 function. (**B** and **C**) Results of RNA quantification 1 (B) and 2 (C) shown in (A); siH1, siRNA targeting RNase H1; siNTC2, nontargeting siRNA; UTC, nontreated cells. *P* < 0.0001 (****) was calculated relative to the siNTC2 by one-way ANOVA (B). Nonsignificant (ns) was calculated by unpaired *t*-test (C).

To understand the mechanism of *FXN*-activating ASOs, we sought to establish whether S10- and S30-derived ASOs activated *FXN* expression by altering local R-loop dynamics. RNase H1 is the major enzyme that resolves R-loops in mammalian cells (37). To test whether the *FXN* activation by S10- and S30-derived ASOs was dependent on RNase H1, we first knocked down RNase H1 by a validated siRNA (siH1) in GM03816 cells and then, after 60 h, co-transfected the cells with ASO and additional siRNA (Figure 6A) (38). We achieved over 90% knockdown of RNase H1 (Figure 6B) and observed that both S10- and S30-derived ASOs still significantly activated *FXN* expression in this context (Figure 6C). Thus, *FXN* activation by S10- and S30-derived ASOs was independent of RNase H1 function. This suggests that S10- and S30-derived ASOs do not activate *FXN* expression by modulating local R-loop dynamics to enable RNase H1-mediated turnover of R-loops.

### S10- and S30-derived ASOs induce a transcriptome profile distinct from that of wild-type cells

**Figure 7.**
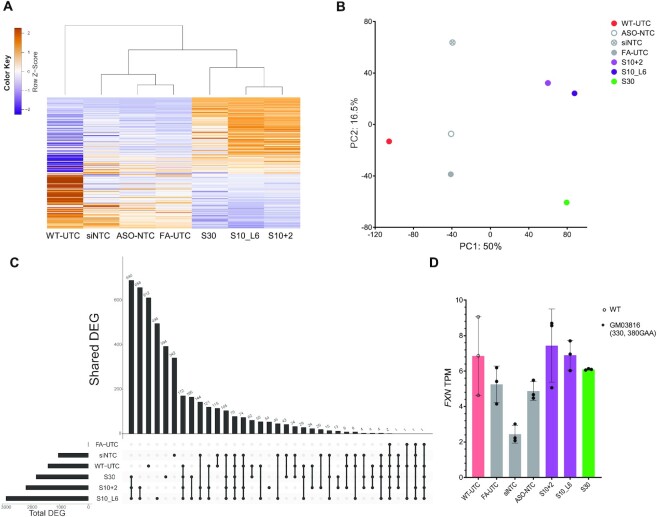
Distinct transcriptome profile induced by S10- and S30-derived ASOs. (**A**) Heatmap of top 200 DEGs relative to ASO-NTC. (**B**) Principal component plot of first two principal components after analysis of genes expressed (TPM > 5) in all samples. (**C**) Upset plot in which the lower panel indicates groups of samples sharing DEGs, and the upper panel indicates the corresponding number of shared DEGs. (**D**) *FXN* mRNA expression levels (TPM) across different treatments.

To understand transcriptome-wide changes after treatment with *FXN*-activating ASOs, we conducted RNA-seq in both GM03816 and WT fibroblasts. For each treatment group, we identified differentially expressed genes (DEGs) relative to the ASO-NTC treated cells. Focusing on the top 200 DEGs (i.e. the expression changes with the smallest *P*-values), we observed that cells treated with S10- and S30-derived ASOs clustered together, with a pattern of gene expression changes distinct from that of WT-UTC (Figure 7A, Supplementary Figure S4 and Table S4). DEGs shared by these three groups showed similar patterns in both the direction and magnitude of gene expression changes relative to all other conditions. Similarly, principal component analysis showed distinct clustering of ASO-treated cells, control conditions and WT-UTC, with 50% of variance (PC1) in gene expression levels explained by differences between these three groups (Figure 7B). Together these analyses indicate that these three ASOs induce a similar transcriptomic profile, and that this shared profile is unique from that of WT-UTC.

To study this in greater detail, we looked at specific DEG changes shared between groups (upset plot, Figure 7C). Cells treated with S10+2, S10_L6 or S30 ASOs showed 690 shared DEGs, the largest number among all groups (Figure 7C). Relative to ASO-NTC, there were relatively few DEGs in the untreated FA cells (FA-UTC) group but over 1000 DEGs in the siNTC group. This suggests that ASO-NTC is a better control maintaining a transcriptome profile more like that of the untreated control (Figure 7B), which is also consistent with the RT-qPCR data (Figure 3D).

Cells treated with S10+2, S10_L6 and S30 showed a trend toward increased *FXN* mRNA expression levels (transcripts per million (TPM), Figure 7D). While there are no statistically significant differences compared to cells treated with ASO-NTC, which is largely due to the intrinsic variance and low expression level of *FXN*, the trends in the RNA-seq data are consistent with the *FXN* up-regulation observed using the QuantiGene and RT-qPCR assays.

In summary, while S10+2, S10_L6 and S30 treatments appear to increase *FXN* mRNA expression in patient-derived fibroblasts, they do not induce a transcriptomic profile resembling that of WT fibroblasts. This suggests that the global transcriptome-wide changes induced by S10- and S30-derived ASOs might not be downstream of FXN activation. This in turn made us question whether the ASOs were operating through an on-target mechanism.

### *FXN* activation by S10- and S30-derived ASOs is mediated by an indirect effect in 293T cells

**Figure 8.**
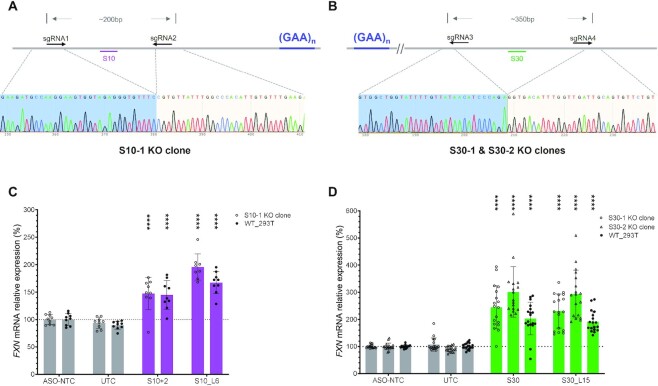
*FXN* activation by S10- and S30-derived ASOs is mediated by indirect effects. (**A** and **B**) Sequencing confirms removal of the S10 (A) and S30 (B) targeting site. (**C** and **D**) *FXN* expression after ASO treatment in WT-293T, S10 KO clone (C) and S30 KO clones (D). sgRNA, single guide RNA used to create the deletion mutants. *P* < 0.0001 (****) was calculated relative to ASO-NTC by one-way ANOVA.

To further understand the mechanism of *FXN* activation by S10- and S30-derived ASOs, we wanted to establish with certainty whether these were on-target events. We designed a pair of guide RNAs flanking each activation hotspot and electroporated them as Cas9 RNP complexes in cells to delete the ASO-binding sites (Figure 8A and B). We chose 293T cells for this experiment because they can readily form a clonal population after seeding at single cell density. Knockout clones were established from single cells and confirmed by Sanger sequencing. We validated that the removal of ASO-binding sites did not change *FXN* expression level or splicing patterns (Supplementary Figure S3).

We transfected two ASOs targeting each hotspot into the corresponding edited cell line. As such, we transfected S10+2 and S10_L6 into the cell line with the S10 region removed (S10-1 KO clone) and S30 and S30_L15 into two cell lines with the S30 region removed (S30-1 and S30-2 KO clones). To our surprise, we found that S10- and S30-derived ASOs were still able to increase *FXN* mRNA level in both WT 293T cells and knockout clones to a similar extent, demonstrating that S10- and S30-derived ASOs increase *FXN* mRNA level by indirect effects (Figure 8C and D).

### Indirect *FXN* activation is unlikely to be mediated by a mutual hybridization-dependent off-target transcript

**Figure 9.**
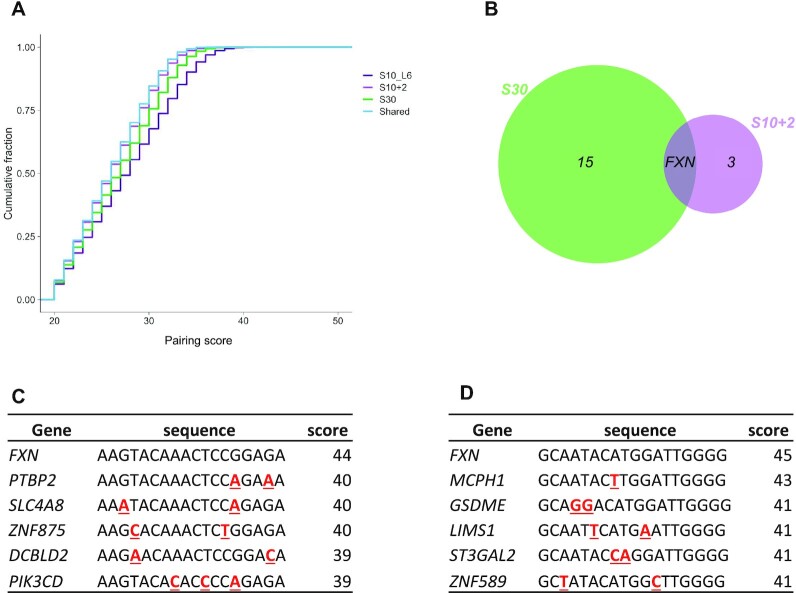
Identifying potential hybridization-dependent off-target transcripts. (**A**) Cumulative distributions (*y*-axis) of pairing scores for matching sites within DEGs in each category (colors). Given a pairing score on the *x*-axis, the value on the *y*-axis indicates the fraction of genes that are not considered matches by this pairing score cutoff. (**B**) Venn diagram showing the number of matched genes with pairing score ≥40. *FXN* was the only shared gene. (**C** and **D**) Top 6 genes with S10+2 (C) and S30 (D) sequence matches, ranked by the pairing score. In all cases, the ASO is antisense to the RNA, but alignments are shown in the ASO orientation. Mismatched nucleotides relative to the ASO are shown in red, bold and underlined.

To identify potential hybridization-dependent off-target sites within expressed genes, we developed a pairing score that upweights complementary matches between the ASO and target sequence while variably penalizing for mismatches depending on the base pair composition (Materials and Methods, Supplementary Table S5). Using this pairing score, we identified potential ASO-binding sites (Figure 9A and B) by scanning all expressed genes for pairing sites and calling any site with a pairing score ≥40 as a candidate match. We found that S10+2 and S30 had 3 and 15 unique potential off-target sites, respectively, and that *FXN* was the only mutual hybridization-dependent target (Figure 9B–D), with the highest possible pairing score for both ASOs. Notably, only 3 out of these 18 potential off-target transcripts were differentially expressed in the RNA-seq data (Supplementary Table S5). This suggested that the indirect activation of *FXN* expression was unlikely to be mediated by silencing or activation of an off-target transcript with complementary sites to both S10+2 and S30.

The fact that we observed no common off-target transcripts, despite similar transcriptomic profiles observed for all three ASOs (Figure 7A–C), suggests two potential hypotheses for *FXN* activation with ASO treatment. (i) The ASOs at the S10 and S30 hotspots bind and regulate distinct off-target transcripts but induce similar overall downstream effects on the gene expression profile of cells; or (ii) the active ASOs regulate gene expression through a hybridization-independent interaction. To explore the likelihood of these hypotheses, we carried out two additional experiments: changing the backbone chemistry and conducting a series of mismatch studies.

### PMO analogs of active ASOs do not activate *FXN* expression

Nonhybridization-mediated effects are often driven by protein binding; they can be specific to one type of backbone and are often associated with the high protein binding of PS-modified ASOs (39,40). PMO chemistry is neutral and nonimmunostimulatory (41,42). For applications in splice-switching, overlapping ASOs of PS and PMO chemistries have both been seen to be active. For example, two ASOs of similar sequence used for applications in dystrophin exon skipping are drisapersen (Kyndrisa, PS-2′OMe-RNA, reached Phase 3 trials) and eteplirsen (Exondys 51, PMO, approved) (43,44). Thus, the comparison of activity of PS and PMO backbones for steric blocker applications might provide additional evidence as to whether a given phenomenon is driven by hybridization or not.

We purchased PMO analogs of S10 and S30 and transfected them into 293T cells using the Endo-Porter reagent at 5 and 10 μM concentrations. We observed that the PMO analogs were unable to activate *FXN* expression (Supplementary Figure S5). Because PMO compounds have significantly lower protein binding relative to PS-backbone ASOs (45–47), this is consistent with the idea that the activation we observed may be specific to the PS backbone and may not be driven by hypothesis (i), i.e. ASOs binding to distinct off-target transcripts but inducing similar overall downstream effects on the gene expression profile of cells.

### Mismatch studies identify guanosine-rich motifs required for *FXN* activation in active ASOs

Certain motifs within ASOs have been observed to correlate with toxicity or stress responses in a nonhybridization-mediated manner. These include, for example, TGC and TCC (48), 3′-terminal guanosines (49), CG dinucleotides (50,51) and G-quadruplex (G_4_) (52–58). Toxic ASOs have been observed to show generally higher levels of protein binding than nontoxic ASOs (48,59). The existence of a toxic or stress-inducing motif in our ASOs might suggest a mechanism consistent with hypothesis (ii), i.e. the active ASOs regulate gene expression through a hybridization-independent interaction. We therefore carried out a mismatch study to explore whether we could identify such a motif in the active ASOs in this study.

**Figure 10.**
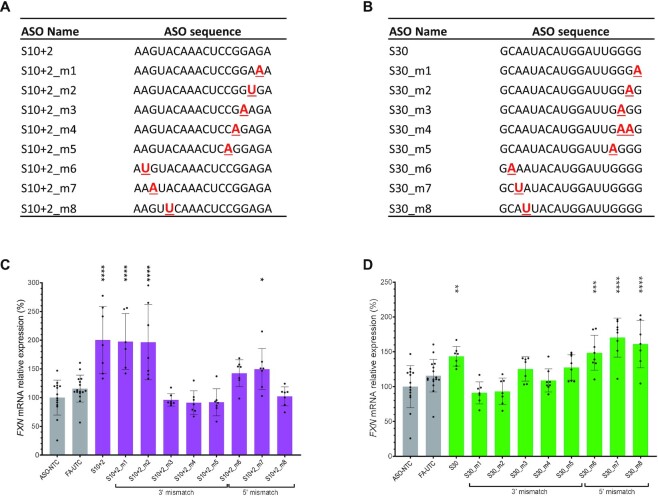
*FXN* activation by S10- and S30-derived ASOs is dependent on key motifs. (**A** and **B**) Names and sequences of ASOs used in this mismatch study (S10-derived ASOs, (A); S30-derived ASOs, (B)). All ASOs have full PS backbones and full 2′-MOE modifications. Mismatched nucleotides relative to the parent ASO are shown in red, bold and underlined. (**C** and **D**) *FXN* mRNA quantification after GM03816 cells were treated with mismatched S10- (C) and S30-derived ASOs (D).

We designed ASOs carrying mismatches in various positions within S10+2 and S30 (Figure 10A and B). We synthesized and purified these ASOs, and tested their ability to activate *FXN* expression in GM03816 patient fibroblasts. For S30, we identified an essential G_4_ motif at the 3′-end, while mismatches at the 5′-end of S30 still maintained *FXN* activation (Figure 9D). For S10+2, interestingly, there seemed to be two essential motifs required for activation, with one of them being ‘CCGG’ (Figure 9C). A single mismatch within either of these regions (S10+2_m3,4,5,8) was sufficient to abolish activation. As such, across both sequences we observed strong sensitivity to a single mismatch in key regions, while mismatches in other regions maintained full activity. This is consistent with the idea that *FXN* activation might be induced by nonspecific protein binding to key motifs within S10- and S30-derived ASOs.

We examined the sequences we originally screened (Supplementary Table S2) and observed that a number of other ASOs (e.g. S3, S8, S23, G36, and some of the other oligonucleotides from the S10 and S30 microwalk, Figure 2) also contained similar guanosine-rich motifs but failed to activate *FXN* expression. Therefore, additional factors such as specific flanking sequences or secondary structures must be required for the indirect *FXN* activation exerted by S10 and S30 ASOs, the mechanism of which is yet to be fully understood.

## DISCUSSION

With the benefit of advanced chemical modification and ligand conjugation approaches, oligonucleotide drugs have been successful in the liver and central nervous system (4). However, most approved oligonucleotide therapies either knock down their RNA target or modulate pre-mRNA splicing. There are huge unmet medical needs in diseases caused by insufficient expression of a specific gene, including FA and the many diseases of haploinsufficiency. Technology that enables robust and consistent gene activation would be transformative. As noted in the Introduction, several encouraging oligonucleotide-induced gene activation approaches have been published in recent years (5–17), but the generality of these approaches remains unclear.

### Gene activation by oligonucleotides is challenging and vulnerable to normalization artifacts

It is fairly easy to achieve over 80% knockdown by siRNAs or gapmer ASOs, while only 2- to 3-fold gene activation was achieved in most of the above oligonucleotide-induced gene activation studies. Therefore, activation studies are inherently vulnerable to normalization artifacts of two types. The first normalization artifact is that nonoptimal NTCs might lower the expression of the target gene, making other ASOs seem to have an apparently higher level of target gene expression after normalization. To avoid this, we suggest including multiple NTCs as well as untreated controls, and ensuring that the NTC and untreated controls show similar gene expression levels. The second potential normalization artifact is that the ASO might interfere with the expression of a housekeeping gene. For example, if an ASO could decrease by 50% the expression of a housekeeping gene, it would seem to show a 2-fold increase in target gene expression after normalization by this housekeeping gene. Validating gene expression through normalization by multiple housekeeping genes or quantitating activation by RNA-Seq can mitigate this risk.

### Mechanisms, motifs and backbones

Our study observed nonspecific gene activation using steric blocker ASOs, which are widely considered to have fewer off-target effects relative to gapmer ASOs. The potential for this class of compounds to induce hybridization-mediated effects has recently been highlighted (60), and our work underlines that they are also vulnerable to nonhybridization-mediated effects.

Similarly, other authors have observed that ASOs containing G4-motifs (particularly in the context of PS backbone modification) have a high risk of inducing unintended changes in gene expression (50–58), but our work underlines that similar changes (as assessed by transcriptome-wide changes in RNA levels) can be induced by other G-rich motifs (such as CCGG in S10). Both G-rich motifs we observed were context dependent, as multiple ASOs but not all ASOs containing these motifs showed the off-target *FXN* activation (Figure 2B and E).

We observed that PMO analogs of the two lead sequences did not activate *FXN* expression. Given that PMO oligonucleotides are known to have lower binding to a variety of proteins relative to PS oligonucleotides (45–47), this observation is consistent with the hypothesis that the *FXN* activation we observe is more likely to be driven by off-target protein interactions than by base pairing. Testing PMO analogs can be a straightforward validation tool to help differentiate hybridization-mediated effects from backbone-induced off-target effects for future work on novel mechanisms as well as other contexts.

### Gene editing as a means to differentiate direct and indirect effects in gene activation and silencing

Besides avoiding the two normalization artifacts above, target validation is also important. Our ASO screen and subsequent micro-walk identified two unique targeting hotspots for *FXN* activation, suggesting to us that *FXN*-activation by S10- and S30-derived ASOs were likely on-target events. However, when we deleted the target sites from the genome of 293T cells and the activation was maintained, this showed conclusively that both S10 and S30 activated *FXN* expression by indirect effects. Subsequent work in identifying shared partially complementary transcripts, PS-backbone dependence and mismatch studies suggested that *FXN* activation was less likely mediated by ASO binding to a mutual off-target transcript but more likely driven by G-rich motifs within S10- and S30-derived ASOs. Li *et al.* previously used genome editing to demonstrate the on-target nature of a small RNA-mediated activation effect (61). Thus, our work, combined with that of Li *et al.* (61), highlights genome editing as an excellent method to distinguish direct and indirect effects in oligonucleotide-mediated gene activation studies.

Gene editing has also proven useful in demonstrating off-target effects previously attributed to RNA interference (RNAi)-mediated gene silencing. For instance, a study demonstrated that previously published RNAi constructs had identical antiproliferative effects in WT cancer cell lines as in clones with the supposed RNAi target genes knocked out, indicating that the off-target effects of RNAi can lead to misidentification of drug targets (62,63).

A decade ago, it would not have been practical to routinely carry out ASO validation by removing the targeting site from the genome. However, with the advent of CRISPR genome editing technologies, this is now within reach of most biomedical research laboratories. Particularly, when ASO mechanisms are novel or the target gene expression change is small, our study shows the value of gene editing to test whether the ASO is acting through an on-target mechanism. We note that some ASO target sites may not be appropriate for this approach, for example, if mutation of the target site alters the original gene expression or splicing.

### Therapeutic perspectives

The *FXN*-activating ASOs identified in this study are unlikely to be therapeutically useful in their current forms. However, future efforts in deepening our understanding of how these ASOs activate *FXN* expression, perhaps via protein binding, might yield valuable knowledge about *FXN* gene expression regulation. Novel therapeutic targets might therefore be identified. Of course, if the ASO-induced *FXN* activation we observed was driven by pathways involving the innate immune response or other stress-related pathways, the targets identified might not yield drugs with a sufficiently clean therapeutic index.

Our work does highlight potential for *FXN* activation by indirect mechanisms. Thus, genome-wide CRISPR or RNAi screening might be a more powerful strategy to identify therapeutically promising targets for *FXN* activation (Minggang Fang and Michael Green, manuscript in preparation).

### Summary and moving forward

In this work, we identified multiple hits at two nonoverlapping hotspots within intron 1 of *FXN* pre-mRNA, compared with multiple negative control oligonucleotides. We carefully verified that the *FXN* activation was not a normalization artifact. These controls and precautions, while essential (64), did not reveal that our compounds were working through an off-target pathway until we carried out the ultimate control of deleting the target site from within cells. Our work highlights the long-known risk of off-target activity by oligonucleotide therapeutics. Thorough use of controls, including genome editing, can minimize the risk of advancing a false positive compound into further stages of development and thus improve the success rate in the development of oligonucleotide drugs for target gene activation.

## DATA AVAILABILITY

Data is available on the Gene Expression Omnibus under accession ID GSE205526 (https://www.ncbi.nlm.nih.gov/geo/). To find potential off-target sites, we used a custom python script available at https://doi.org/10.5281/zenodo.7262358.

## Supplementary Material

gkac1108_Supplemental_Files
