# Supplementary material for: G-rich motifs within phosphorothioate-based antisense oligonucleotides (ASOs) drive activation of FXN expression through indirect effects
Source: Nucleic Acids Res. 2022 Dec 13;50(22):12657–73. doi: 10.1093/nar/gkac1108 (PMC9825156; doi:10.1093/nar/gkac1108)
Supplement: gkac1108_Supplemental_Files [file gkac1108_supplemental_files.zip › FA paper supplementary information_as revised resubmitted.pdf]

Supplementary information

G-rich motifs within phosphorothioate-based antisense oligonucleotides (ASOs) drive activation of *FXN* expression through indirect effects

Feng Wang[1], Ezequiel Calvo-Roitberg[1], Julia M. Rembetsy-Brown[1], Minggang Fang[2], Jacquelyn Sousa[1], Zachary J. Kartje[1], Pranathi Meda Krishnamurthy[1], Jonathan Lee[1], Michael R. Green[2], Athma A. Pai[1], Jonathan K. Watts[1,3]

1. RNA Therapeutics Institute, University of Massachusetts Chan Medical School, Worcester, MA, USA
2. Department of Molecular, Cell and Cancer Biology, University of Massachusetts Chan Medical School, Worcester, MA, USA
3. Department of Biochemistry and Molecular Biotechnology, University of Massachusetts Chan Medical School, Worcester, MA, USA

Fig S1

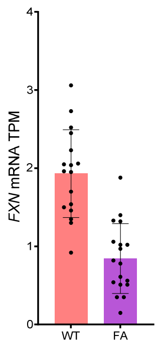

Figure S1. *FXN* expression across in fibroblast cell lines derived from 18 FA patients and 17 unaffected control individuals (1). We and others also observe inherent variability in the expression of *FXN* within a given cell line in culture (1–7).

Fig S2

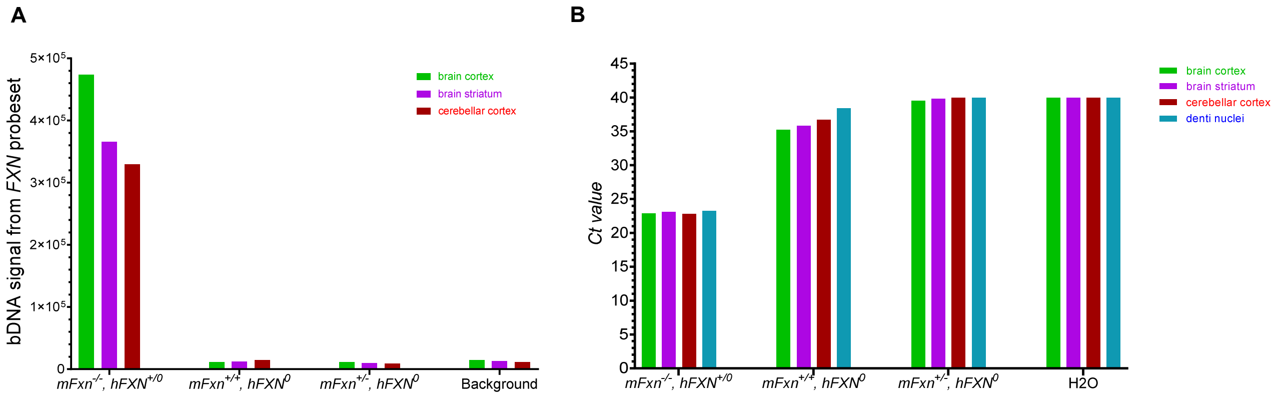

Figure S2. *FXN* bDNA probe set and qPCR primers are specific to human *FXN*. (**A-B**) bDNA signal from *FXN* probe set (A) and Ct value from *FXN* qPCR primers (B) detecting mouse brain tissues from various genetic backgrounds. *mFxn*: mouse *Fxn*. *hFXN*: human *FXN*.

Fig S3

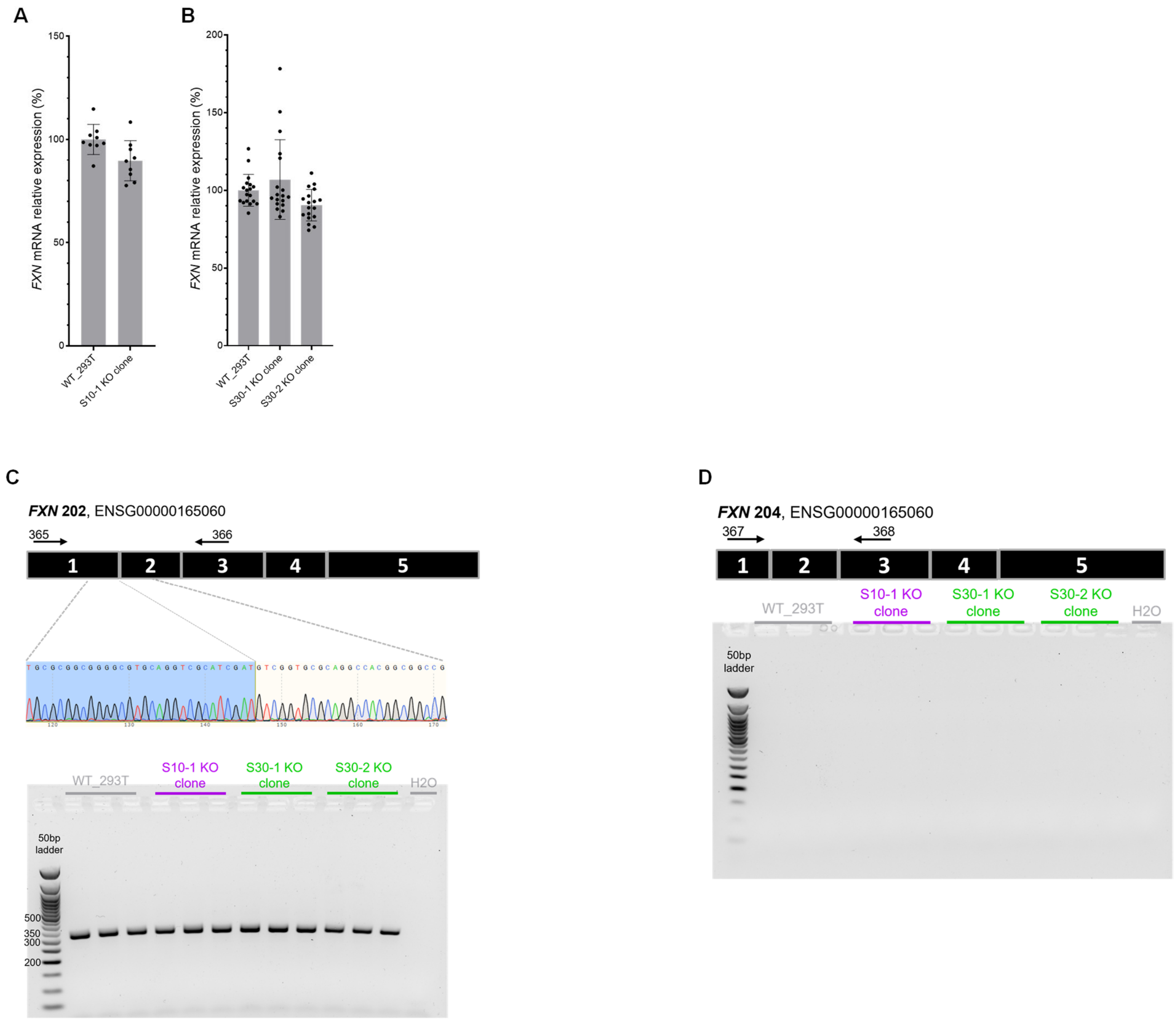

Figure S3. Removal of S10 or S30 binding sites by CRISPR/Cas9 does not change *FXN* expression or splicing. (**A-B**) *FXN* mRNA is at similar levels between WT_293T and knockout clones removing S10 binding site (A) or S30 binding site (B). (**C-D**) *FXN* 202 (C) and *FXN* 204 (D) isoform mRNAs were detected by RT-PCR. Numbered black boxes represent exons. *FXN* 202 and *FXN* 204 have unique first exons.

Fig S4

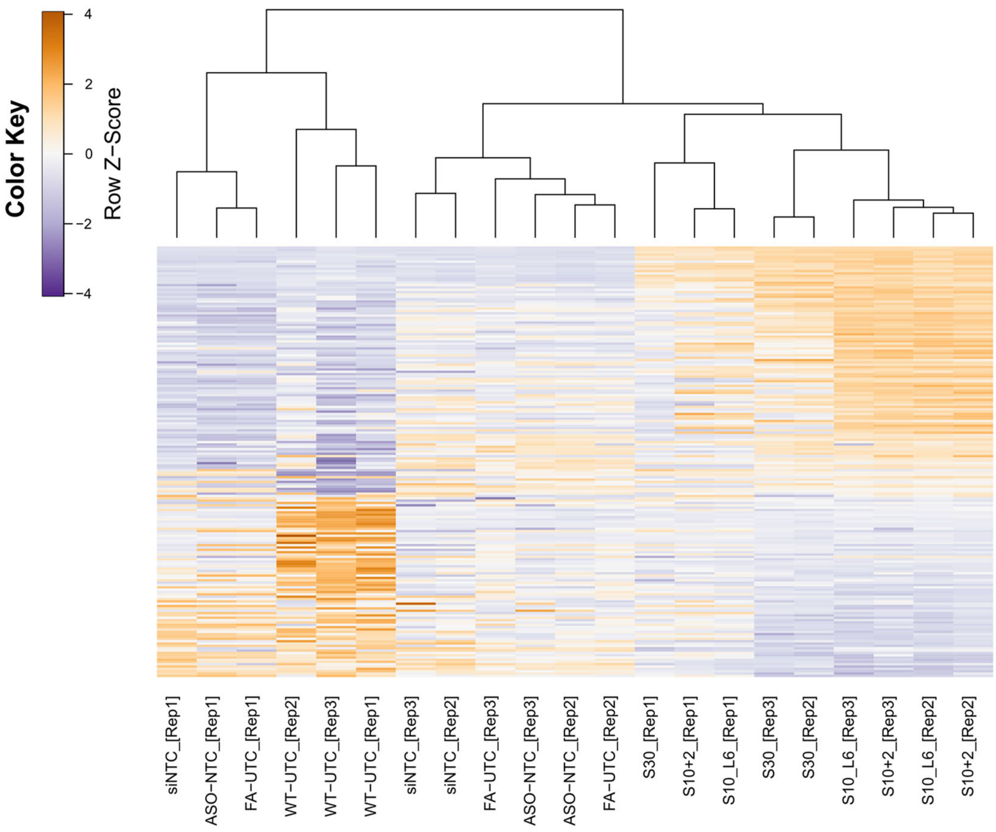

Figure S4. (**A**) Heatmap based on replicates.

Fig S5

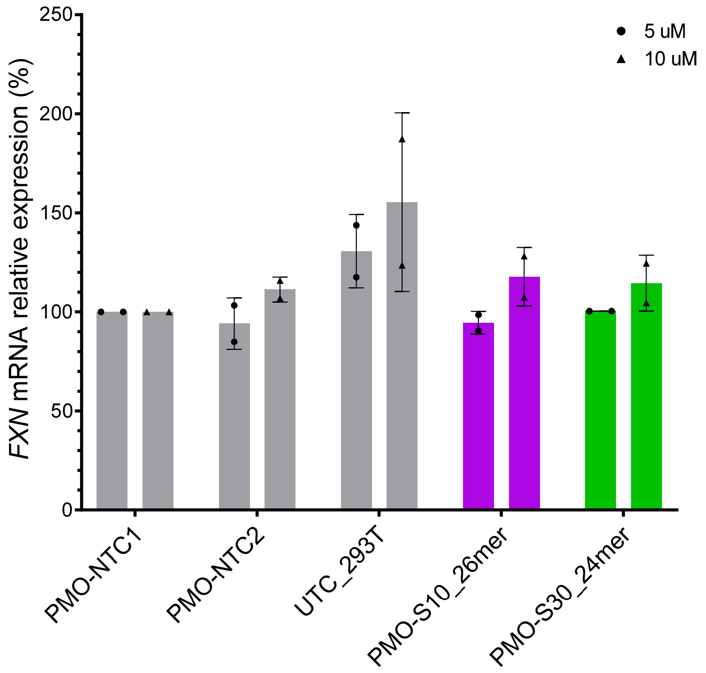

Figure S5. Test of PMO analogues of S10- and S30-derived ASOs in 293T cells.

Table S1. Off-target genes of GAA-repeat-targeted oligonucleotides

Off-target genes	(GAA)n/(AAG)n/(AGA)n	Protein coding	Binding site	Pairing strand
C18orf25	6	Y	Exon	Template strand
EXOC6B, mRNA variants 1-8	7	Y	Exon	Template strand
PRDM10, mRNA variants 1-4	10	Y	Exon	Template strand
RIN2	22/6	Y	Intron/Exon	Template strand
FOXP2	6	Y	Exon	Template strand
ERCC1	6	Y	Exon	Template strand
CDC42	6	Y	Exon	Template strand
DLG2	7/8	Y	Exon/Intron	Template strand
TMPRSS4	10	Y	Exon	Template strand
KPNA6	6	Y	Exon	Template strand
SOX9	6	Y	Exon	Template strand
CD3EAP/POLR1G	6	Y	Exon	Coding strand
DKC1, mRNA variants 1-6	7	Y	Exon	Coding strand
FAM177A1, mRNA variants 1-3	7	Y	Exon	Coding strand
FCN1, mRNA	19	Y	Exon	Coding strand
KAT6B, mRNA variants 1-3	8	Y	Exon	Coding strand
KIAA0040, mRNA variants 1-6	10	Y	Exon	Coding strand
MAT1A	6	Y	Exon	Coding strand
MLEC	6	Y	Exon	Coding strand
NEXN-AS1, long non-coding RNA	32	N	ncRNA	Coding strand
NFIC	6	Y	Exon	Coding strand
OTOF	6	Y	Exon	Coding strand
PRRG4, mRNA	7	Y	Exon	Coding strand
RABL6	6	Y	Exon	Coding strand
RPS6KA5	8	Y	Exon	Coding strand
TECPR2	6	Y	Exon	Coding strand
TERF2IP	6	Y	Exon	Coding strand
TMIE, mRNA	9	Y	Exon	Coding strand
TGM5	7	Y	Intron	Coding strand

The following tables are provided as separate supporting files.

Table S2. Oligonucleotide list

Table S3. List of sgRNAs, primers, and bDNA probes

Table S4. Differentially expressed gene (DEG) list

Table S5. ASO pairing score matching results

Supplementary references:

1. Napierala,J.S., Li,Y., Lu,Y., Lin,K., Hauser,L.A., Lynch,D.R. and Napierala,M. (2017) Comprehensive analysis of gene expression patterns in Friedreich's ataxia fibroblasts by RNA sequencing reveals altered levels of protein synthesis factors and solute carriers. *Dis Model Mech*, **10**, 1353–1369.

2. Bon,C., Luffarelli,R., Russo,R., Fortuni,S., Pierattini,B., Santulli,C., Fimiani,C., Persichetti,F., Cotella,D., Mallamaci,A., *et al.* (2019) SINEUP non-coding RNAs rescue defective frataxin expression and activity in a cellular model of Friedreich's Ataxia. *Nucleic Acids Research*, **47**, 10728–10743.

3. Li,L., Matsui,M. and Corey,D.R. (2016) Activating frataxin expression by repeat-targeted nucleic acids. *Nat Commun*, **7**, 10606.

4. Shen,X., Beasley,S., Putman,J.N., Li,Y., Prakash,T.P., Rigo,F., Napierala,M. and Corey,D.R. (2019) Efficient electroporation of neuronal cells using synthetic oligonucleotides: identifying duplex RNA and antisense oligonucleotide activators of human frataxin expression. *RNA*, **25**, 1118–1129.

5. Li,L., Shen,X., Liu,Z., Norrbom,M., Prakash,T.P., O'Reilly,D., Sharma,V.K., Damha,M.J., Watts,J.K., Rigo,F., *et al.* (2018) Activation of Frataxin Protein Expression by Antisense Oligonucleotides Targeting the Mutant Expanded Repeat. *Nucleic Acid Therapeutics*, **28**, 23–33.

6. Shen,X., Wong,J., Prakash,T.P., Rigo,F., Li,Y., Napierala,M. and Corey,D.R. (2020) Progress towards drug discovery for Friedreich's Ataxia: Identifying synthetic oligonucleotides that more potently activate expression of human frataxin protein. *Bioorganic & Medicinal Chemistry*, **28**, 115472.

7. Tomassini,B., Arcuri,G., Fortuni,S., Sandi,C., Ezzatizadeh,V., Casali,C., Condo,I., Malisan,F., Al-Mahdawi,S., Pook,M., *et al.* (2012) Interferon gamma upregulates frataxin and corrects the functional deficits in a Friedreich ataxia model. *Human Molecular Genetics*, **21**, 2855–2861.
